# Stress Modulates Instrumental Learning Performances in Horses (*Equus caballus*) in Interaction with Temperament

**DOI:** 10.1371/journal.pone.0062324

**Published:** 2013-04-23

**Authors:** Mathilde Valenchon, Frédéric Lévy, Armelle Prunier, Chantal Moussu, Ludovic Calandreau, Léa Lansade

**Affiliations:** 1 INRA, UMR85 Physiologie de la Reproduction et des Comportements, Nouzilly, France; 2 CNRS, UMR7247 Physiologie de la Reproduction et des Comportements, Nouzilly, France; 3 Université François Rabelais de Tours, Tours, France; 4 IFCE, Nouzilly, France; 5 INRA, UMR1348 PEGASE, Saint-Gilles, France; 6 Agrocampus Ouest, UMR1348 PEGASE, Rennes, France; Utrecht University, Netherlands

## Abstract

The present study investigates how the temperament of the animal affects the influence of acute stress on the acquisition and reacquisition processes of a learning task. After temperament was assessed, horses were subjected to a stressor before or after the acquisition session of an instrumental task. Eight days later, horses were subjected to a reacquisition session without any stressor. Stress before acquisition tended to enhance the number of successes at the beginning of the acquisition session. Eight days later, during the reacquisition session, contrary to non-stressed animals, horses stressed after acquisition, and, to a lesser extent, horses stressed before acquisition, did not improve their performance between acquisition and reacquisition sessions. Temperament influenced learning performances in stressed horses only. Particularly, locomotor activity improved performances whereas fearfulness impaired them under stressful conditions. Results suggest that direct exposure to a stressor tended to increase acquisition performances, whereas a state of stress induced by the memory of a stressor, because it has been previously associated with the learning context, impaired reacquisition performances. The negative effect of a state of stress on reacquisition performances appeared to be stronger when exposure to the stressor occurred after rather than before the acquisition session. Temperament had an impact on both acquisition and reacquisition processes, but under stressful conditions only. These results suggest that stress is necessary to reveal the influence of temperament on cognitive performances.

## Introduction

The relationship between stress and cognition has been studied extensively in the past few decades (reviewed by [1]–[4]), but little is known about the variability of this phenomenon among individuals. This variability may depend on behavioral characteristics of individuals, such as temperament [5]. In recent years, very few authors have explored these relationships among stress, cognition, and temperament. Among them, Jiao et al. [6] illustrated how stress and dimension of anxiety co-influence learning performances. Rats from two strains (Wistar-Kyoto and Sprague-Dawley) that differed in their anxiety levels were compared in terms of acquisition and extinction of an instrumental task associated with two different intensities of stressors. The authors showed that the most anxious rats extinguished slower the task with the higher intensity of stressor than with the lesser intensity of stressor, whereas there was no effect of the intensity of stressor in less anxious rats. These data suggest that the influence of stress on cognitive performances may differ according to the temperament of individuals. However, the authors typically focused on only one dimension related to anxiety, whereas many other behavioral dimensions characterize an individual.

Therefore, we studied the influences of several dimensions of temperament and stress on learning performances in horses. For this, we used a complete model of temperament developed in this species that does not exist in usual experimental models as rodents. This model characterizes each individual on the basis of five dimensions of temperament that were previously shown as stable over time and across situations [7], [8]: fearfulness, gregariousness, reactivity to humans, level of locomotor activity, and tactile sensitivity [9]–[12]. Also, a better knowledge of the influence of both temperament and stress on horse cognition is of prime interest, since this species is often subjected to cognitive challenges and stressors in both feral and domestic conditions. As an applied perspective, the current study might allow to personalize training conditions according to the temperament of each horse.

To evaluate the influence of stress, horses were exposed to an acute stressor just before or just after the acquisition of the task. Depending on its timing, it should affect preferentially the acquisition or the consolidation processes. We focused on these distinct processes because several authors showed that the influence of stress on learning performances also depends on which stage of memory is involved (reviewed by [13]). For instance, a state of stress when acquisition processes are predominant may enhance (e.g. in humans [14]) or impair performances (e.g. in rodents [15], [16]). Opposite effects are also reported with stress after acquisition in rodents. Indeed, a state of stress when consolidation processes are predominant may either impair [13], [16], [17], have no effect [18], or even potentiate ulterior performances of retrieval or reacquisition [19].

Learning performances in horses were investigated with an instrumental task that consisted of touching a distally indicated cone with its nose to obtain a food reward. This task was chosen due to its difficulty, with the expectation that not all subjects would be successful, thus inducing variability among individuals. This variability is necessary to evaluate the influence of temperament. We have assessed acquisition, retrieval after one week, and reacquisition performances. The overall aims of this research were to determine whether the timing of stress affects learning performances, and whether temperament influences performances differentially according to this stress.

## Materials and Methods

### Animals

Forty-nine female Welsh ponies (age 7±1 years old) were randomly divided into three groups: SB group (stressed before acquisition, N = 15), SA group (stressed after acquisition, N = 15), and NS group (non-stressed, N = 19). These horses were bred together at the experimental unit of the INRA of Nouzilly (National Institute for Agricultural Research, France) and they were accustomed to being handled (regularly haltered and tethered). Before the experiment, the horses lived together outdoors during summer and indoors during winter. During the experimental period, the animals were randomly housed in groups of 3 per small stable (3.5 m×5.4 m) or 10 per large stable (10 m×10 m). The allotment to the small and large stables changed randomly every day, regardless of the experimental group. All horses spent 2 h daily in a large outdoor paddock together (75 m×75 m). They had straw bedding and received concentrated food (pellets) twice a day. No food deprivation was used. Water was available ad libitum.

### Temperament Tests

Before the learning task, we assessed the temperament of each horse. Five dimensions of the horses temperament–fearfulness, gregariousness, reactivity to humans, level of locomotor activity, and tactile sensitivity–were assessed according to the procedures of Lansade et al. [9]–[12] adapted from LeScolan et al., Wolff et al., and Visser et al. [20]–[22].

#### Experimental apparatus

**Figure 1:**
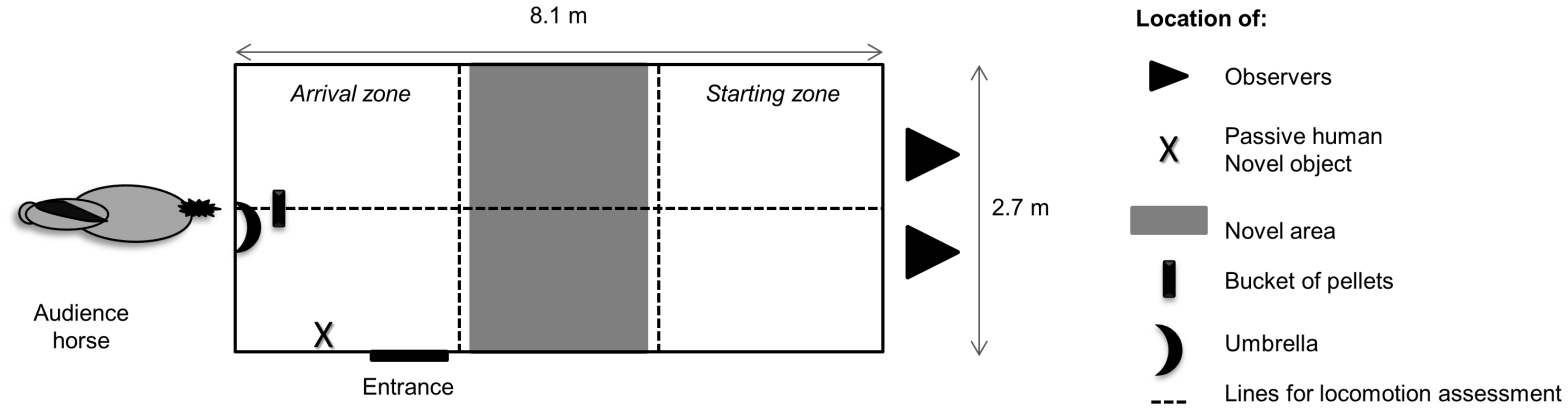
Schematic representation of the experimental apparatus for the temperament tests.

Tests occurred in a box (2.7×8.1 m). Two observers were hidden behind a one-way mirror. A familiar audience horse was attached outside the box on the opposite side. The tested horse could see the audience horse; thus, social isolation was avoided (**Fig. 1**). Six behavioral tests were conducted over a period of approximately 30 min per horse. Each test has been validated to assess one temperament dimension. We recorded behavioral parameters that appear to be reliable indicators of temperament owing to their stability over time and across different situations [9]–[12]. They are indicated at the end of each test procedure.

#### Experimental procedure

After a habituation phase where the horse was free in the loose box for 360 s, the tests occurred exactly in the order presented below:

(1) Passive human test (reactivity to humans): to assess reactivity to humans [9], the experimenter entered the box and stayed motionless for 180 s. We recorded the number of sniffing and nibbling at the experimenter (nibbling is an exploratory behavior where the horse’s jaws are closed and move upward and downward against a support [23]).

(2) Tactile sensitivity test: to measure the dimension of tactile sensitivity [12], the experimenter held the horse and a second experimenter applied a von Frey filament (Stoelting, IL, USA) to the base of the horse’s withers. This filament consists of a hard plastic body connected to a nylon thread. It was calibrated to exert a specific force on the skin, from 0.008 g to 300 g. The filament was applied perpendicularly on the animal’s skin until the nylon filament started to bend. The response was coded in a binary form (trembling/not trembling) according to the reaction of the platysma muscle to the filament application. In the first part of the test, we applied a 0.008-g filament to the right side of the horse and then a 300-g filament on its left side. The same procedure was repeated between the novel area test and the surprise test (explained below), except that we applied filaments of 0.02 g and 1 g respectively. We recorded the number of times the horses responded to the filaments. The most sensitive horses were the ones who responded the most often.

(3) Novel object test (fearfulness): this test assessed the horse’s reactivity to novelty, which is a trait underlying the dimension of fearfulness [10]. An object, unknown to the horse, was placed in the box for 180 s. The object constituted of a horizontal piece of wood (diameter: 0.03 m; length: 1 m) surrounded by a piece of green plastic (height: 0.8 m) and colored filaments. We recorded the numbers of sniffing, nibbling, and glancing at the novel object.

(4) Social isolation test (gregariousness): to assess gregariousness [11], the audience horse was removed from the sight and sound of the tested horse for 90 s and we recorded the number of neighs of the tested horse.

(5) Novel area test (fearfulness): this test also assessed reactivity to novelty, which is a trait underlying the dimension of fearfulness [10]. The floor of the loose box was divided into three zones, each 2.7 m×2.7 m (**Fig. 1**). The first zone was the starting zone (on the right in **Fig. 1**), and the third zone, the arrival zone (on the left in **Fig. 1**). The arrival zone contained a bucket of pellets that the horses were familiar with. Just before the test, the horses underwent a habituation phase during which they learned how to go from the starting zone to the arrival zone. To achieve this, an experimenter led the horse by the halter to the starting zone and released it so that it was free to go to the arrival zone for eating. This procedure was repeated three times. Then, a pink carpet (2 m×2.7 m) was placed in the second zone. As in the habituation stage, the experimenter released the horse into the starting zone and recorded the time taken to cross the carpet. If the horse did not cross the area within 180 s, the test was ended and a time of 181 s was assigned.

(6) Surprise test (fearfulness): this test assessed reactivity to suddenness, which is another trait underlying the dimension of fearfulness [10]. The experimenter, not visible to the horse, opened a black umbrella in front of the animal 3 s after it started eating from the bucket of pellets placed near the entrance (**Fig. 1**). The time taken by the horse to resume eating was recorded. If the horse did not resume eating within 180 s, the experimenter stopped the test and assigned a time of 181 s. We also recorded the flight distance.

In addition, the loose box was virtually divided into six areas of equal size to assess the level of locomotor activity. We recorded the number of areas crossed and the amount of trotting during the habituation phase, passive human test, social isolation test, and novel object test.

Finally, we continuously recorded the number of startled reactions and blowing during the temperament tests (except during tactile sensitivity tests, novel area test, and surprise test, because the durations of these tests varied among horses).

### Learning Task

Horses were trained to touch a target (traffic cone) pointed out by an experimenter using a gestural cue. The task was adapted from Williams et al. [24] and Whistance et al [25]. This task is considered an instrumental task because the horse had to perform an action (touching the cone) under the influence of reinforcement factors (positive reinforcement: food reward) when detecting a particular stimulus (a distal cue given by the experimenter). The horses faced two cones, and at each trial, they had to touch one of the two cones to obtain a food reward according to the experimenter command (a dynamic pointing toward one of the two cones). This two-choice system was chosen after preliminary tests showed that, when a single cone was used, the horse constantly touched the cone or stayed very close to it, rather than paying attention to the experimenter command.

**Figure 2:**
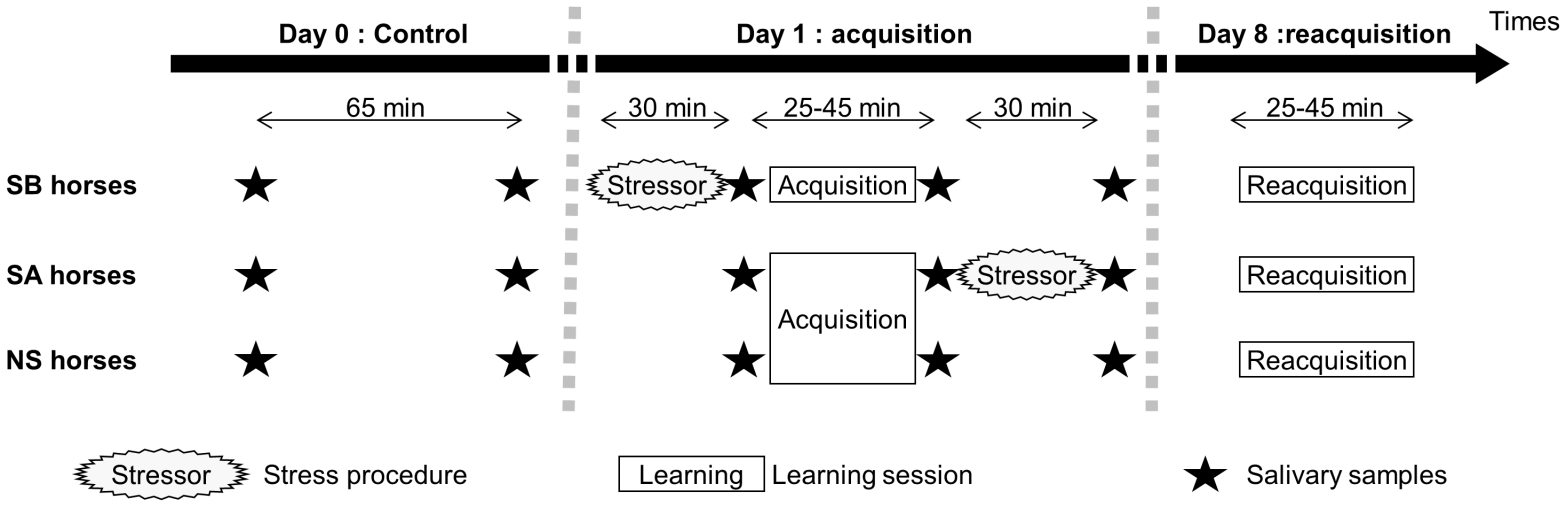
Schematic representation of the experimental protocol for the various groups. These are the SB group: stressed before learning acquisition, the SA group: stressed after learning acquisition, and the NS group: non-stressed.

All the horses were subjected to the learning task on day 1 (acquisition session), and again, eight days later (reacquisition session). On day 1, horses were exposed to an acute stressor immediately before acquisition (stressed before, SB group) or just after acquisition (stressed after, SA group). A third group was not exposed to any acute stressor (non-stressed, NS group) (**Fig. 2**).

#### Learning apparatus

**Figure 3:**
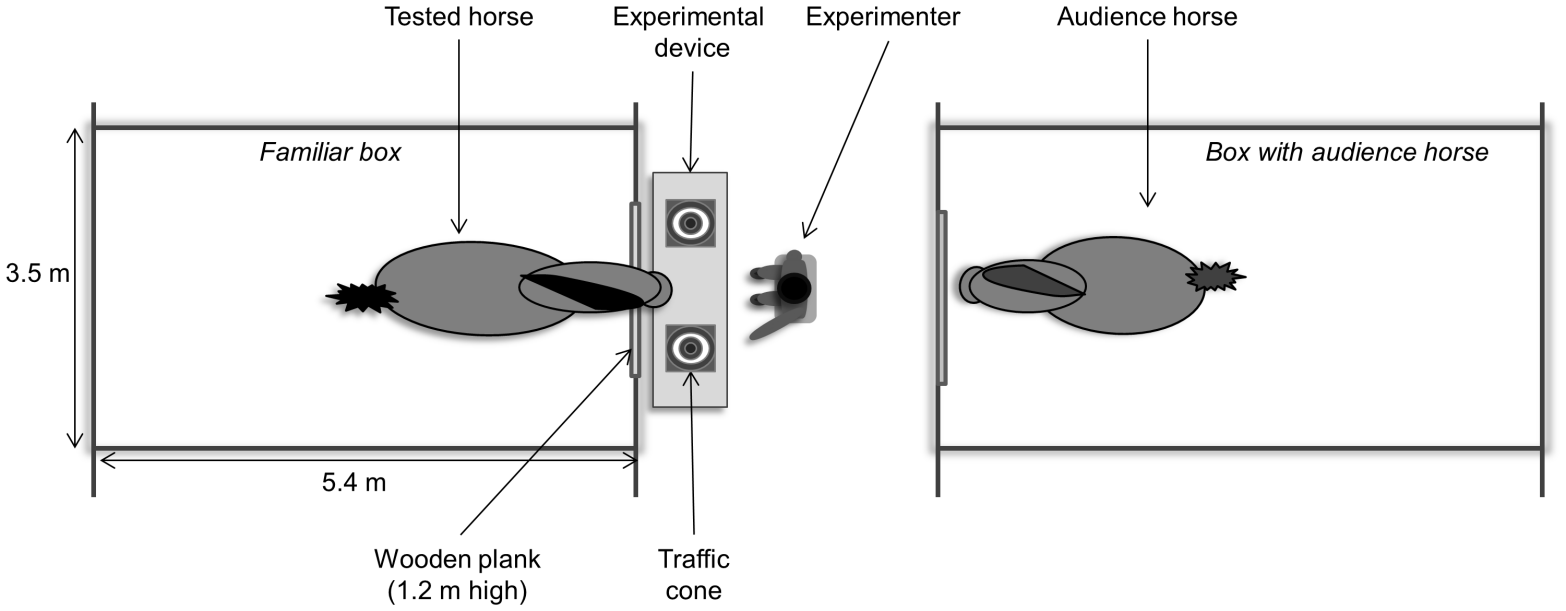
Schematic representation of the learning apparatus. The aim of the task was to touch the cone pointed out by the experimenter (the left or right one at random). An audience horse was placed in front of the tested horse to prevent social isolation.

The animals were individually maintained with two leads (1.2 m long) in a familiar box (3.5 m×5.4 m) such that they were in front of an opened door blocked by a 1.2-m-high wooden plank. The experimenter sat down in front of the horse. Two traffic cones (0.45 m high) separated by 0.4 m were placed on a horizontal wooden plank fixed at a height of 0.3 m above the ground. These traffic cones were placed between the horse and the experimenter such that the horse could touch the cones but not the experimenter (**Fig. 3**). The experimenter was the same adult woman through the learning procedures. The same audience horse as the one used during the temperament tests was placed in a box facing the box where the test was conducted. A similar wooden plank was placed in front of the opened door of the audience horse’s box and made it visible to the tested horse.

#### Familiarization with the learning apparatus

The horses were first familiarized with the learning apparatus and the experimenter. A 30-min long habituation session was conducted, where the horse was placed in front of the cones and was allowed to explore the experimental apparatus. An experimenter offered the horse the opportunity to eat a handful of pellets from his hand twice per session (approximately 7–10 g). Horses were familiarized with the apparatus for 3–5 sessions, until they accepted eating the pellets twice per session from the hand of the experimenter during a session. Forty-three horses met this criterion in the third session, four in the fourth session, and two in the fifth session. The number of familiarization sessions did not differ among the SB, SA, and NS groups (KW, ddl = 2, P>0.10).

#### Learning procedure

Each animal underwent 30 consecutive trials on day 1 (acquisition session) and 30 consecutive trials on day 8 (reacquisition session). Before each trial, the experimenter, who was seated on a chair so he faced the midline of the board between the two cones, (**Fig. 3**), shook a bucket of pellets placed under his chair in order to attract the attention of the tested horse. The trial started as soon as the horse looked at the experimenter or after a maximum of three shakes. Then, the experimenter randomly indicated to one of the two cones (the rewarded cone). The aim of the task was to make the horse touch the rewarded cone by using distal indications to get a food reward. If distal indications did not work, proximal indications were used until it touched the rewarded cone and then received the food reward. Proximal indications were used to guide the horse to make the act of touching the cone, to maintain its motivation, and to equilibrate the quantity of food intake among the horses. The distal and then the proximal indications were presented successively to a horse until it touched the rewarded cone with its nose:

Distal indications: during the distal indications, the distance between the experimenter’s fingers and the cone was between 0.5 m and 1 m. The experimenter pointed out the cone, moving his forefinger up and down (7 times in 10 s). If the horse did not touch the rewarded cone, he repeated the action with his forearm the same number of times.
Proximal indications: during the proximal indications, the distance between the experimenter’s fingers and the cone was between 0 m and 0.03 m. The experimenter pointed out the cone by moving his entire arm up and down (7 times in 10 s). If the horse did not touch the cone, he tapped the top of the cone with his finger the same number of times.

If the horse did not touch the rewarded cone during proximal indications, the experimenter showed the food to the horse for a maximum of 30 s and tried to attract it toward the cone until it touched it. If the horse still did not touch the reward cone, the trial was ended.

The food reward was always given to the horse in the experimenter’s hand at equal distance from the two cones so the reinforcement was given independently of the side of the cone touched. Trials were separated by 10 s. For each trial, we recorded if the horse touched a cone at the time the experimenter made distal (“distal response”) or proximal indications (“proximal response”), and if this cone was the rewarded cone or not. A “success” was defined as a distal response toward the rewarded cone. During preliminary studies, the number of successes was the only variable that increased over time in enough horses, so we chose this variable to represent learning ability.

During the 10-s inter-trial intervals, we recorded the occurrence of exploring (sniffing and nibbling) the cone, snorting, neighing, and blowing, as well as the presence or absence of alert posture in each session.

### Stress Procedure

Animals from the SB and SA groups were led one by one with a halter by a second experimenter and were isolated in an unfamiliar test box (2.7 m×2.7 m), 50 m away from the stable where the learning sessions occurred. Two tarpaulins and two white sheets were hung up against the box walls. During a 30-min period, each horse was submitted to 20 unpredictable sudden events with intervals that randomly varied between 30 s and 90 s. The stressors consisted of an alternation of various stimulations randomly distributed: 6 loud sounds (e.g., dog barks, bell ringing, people talking loudly), 4 aversive sensory stimuli (water jet or air puff emitted toward the horse), 5 sudden movements (shaking of one tarpaulin or of one sheet), and 5 introductions of an unknown object (e.g. colorful cardboard box, colorful balloons). The animals were prevented from seeing the experimenters during this procedure. After this stress procedure, the horses were immediately led by the same experimenter to the learning box (SB horses) or to the home box (SA horses).

### Cortisol Measurement

We assessed salivary cortisol concentration because it is a relevant and non-invasive indicator of physiological stress in horses [26]–[31]. On days 0 and 1, salivary samples were collected with Salivette® Cortisol (SARSTEDT France). On day 0, a day before the experiment, two control samples were collected at an interval of 65 min (**Fig. 2**). On day 1, salivary samples were collected immediately before and after, and 30 min after the acquisition session. The first control sample on day 0 was taken at the same hour as the first sample on day 1, and the second at the same hour as the last sample on day 1. Cotton buds were centrifuged at 3000 g for 20 min at 4°C, and the saliva was stored at −20°C until analysis. Saliva was collected and cortisol was measured in 20-µl samples by using a luminescence immunoassay kit (LIA, IBL, Hamburg, Germany). The measurements were performed without replicates in a single assay. The intra-assay coefficients of variation were 4.8% and 4.1% at 1.8 ng/ml and 9.7 ng/ml, respectively. The assay sensitivity was 0.25 ng/ml. The basal cortisol level was determined by averaging the levels in the 2 saliva samples collected from each horse on day 0.

### Data Analyses

Because of the lack of normality in the data, all behavioral and physiological parameters were analyzed using non-parametric tests. Results are expressed as percentages, or as median and interquartile ranges in the form “M = (1^st^ interquartile–3^rd^ interquartile)” in the text. Both the acquisition and reacquisition sessions were divided into three blocks of 10 trials.

#### Intragroup comparisons

Intragroup comparisons were made using Wilcoxon signed-rank tests for dependent samples (W) or two-tailed Friedman tests (F) followed by Wilcoxon signed-rank tests for dependent samples (W), when more than two variables were compared. Intragroup comparisons of proportions were made using the McNemar tests (McNemar).

#### Intergroup comparisons

Intergroup comparisons were made using two-tailed Mann-Whitney tests (MW, when two groups were compared) or two-tailed Kruskal-Wallis tests (KW, when three groups were compared) followed by Dunn tests (Dunn). Intergroup comparisons of proportions were made using 2I tests. The 2I test is derived from the chi-square test, but it allows the regrouping of experimental treatment [32], [33]. Comparisons of proportions of horses expressing a certain behavior was made when this behavior was expressed by less than 20% of the horses in only one of the groups (startled reactions, snorting, and blowing during the acquisition session, and snorting and blowing during the reacquisition session). When a behavior was expressed by less than 20% of the horses in all of the compared groups, it was not analyzed (neighing during the acquisition and reacquisition sessions, startled reactions during the reacquisition session).

#### Correlations tests

Correlations between temperament data and learning performances were assessed using Spearman rank correlations tests. To avoid repeating these tests excessively, we chose only one global variable representing learning performance: the number of successes (distal responses toward the rewarded cone) for each entire session (acquisition or reacquisition).

Statistical analyses were performed using the statistical package XLSTAT (Addinsoft Inc., France). The level of statistical significance was set at P≤0.05, and the level of tendency at P≤0.10.

### Ethics Statement

The experiments reported in this paper were conducted under a license from the French Ministry of Agriculture (no. 37–125). They only included behavioral observations and non-invasive contact with the horses that did not require the approval of an ethics committee. A minimal number of animals per group was used to statistically test differences. Neither injury nor pain was observed in the horses. The acute stressors used were inspired by real stressors often encountered by domestic animals: short-term social isolation combined with fearful events. In addition, the horses were exposed to each stressor only once, during a 30-min period. The horses belong to the experimental unit “UEPAO” of the INRA of Nouzilly. The owners gave permission for their animals to be used in this study. Horses lived in social groups and were taken to a paddock daily. During the experimental period, no food restriction was used, and during the learning task, only positive reinforcements were used. At the end of the experiment, the animals returned to their normal breeding at the INRA unit.

## Results

### Cortisol Measurements

**Figure 4:**
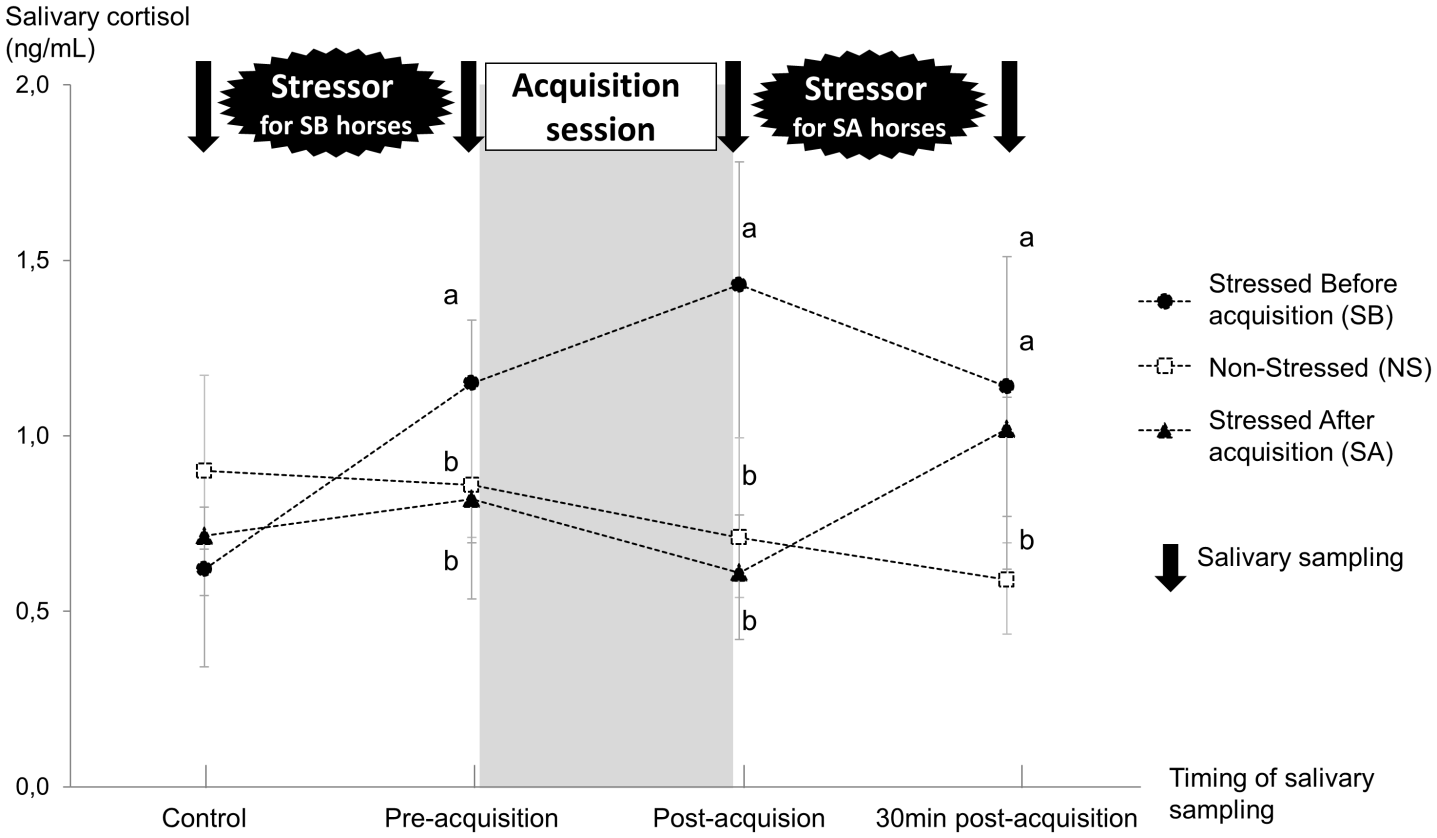
Concentrations of salivary cortisol across the learning task (median ± interquartile). Cortisol concentrations (control) did not differ among the SB (stressed before acquisition session), the NS (non-stressed), and the SA horses (stressed after acquisition session). Pre-acquisition and post-acquisition cortisol concentrations were higher in the SB horses than in the SA and NS horses. Thirty minutes after the end of the acquisition session, cortisol concentrations of the SB and SA horses were significantly higher than cortisol concentration of the NS horses. Difference between groups: a vs. b, P<0.05, Kruskal-Wallis test followed by Dunn tests.

No significant difference was observed in cortisol concentrations on day 0 (control day) between the SB, SA, and NS groups (SB: Stressed Before acquisition session, SA: Stressed After acquisition session, NS: Non Stressed, KW, K = 2.3, P>0.10, **Fig. 4**). On day 1, at the sampling realized both just before and just after the acquisition session, the SB group showed higher cortisol concentrations than the NS (Dunn, P<0.05) and SA groups (Dunn, P<0.05), and the NS and SA groups did not differ (Dunn, P>0.10, KW, ddl = 2, K_pre-acquisition_ = 10.5, P_pre-acquisition_ <0.005, K_post-acquisition_ = 21.7, P_post-acquisition_ <0.0001). At the sampling realized 30 min after the end of the acquisition session, the cortisol concentration in the NS group was significantly lower than that in the SB (Dunn, P<0.05) and SA groups (Dunn, P<0.05). Concentrations in the SB and SA groups did not differ (Dunn, P>0.10, KW, ddl = 2, K = 16.5, P<0.0001). Overall, salivary cortisol increased from about 0.6 ng/ml to 1.15 ng/ml ( = 80% increase in the SB group) or 1.0 ng/ml ( = 67% increase in the SA group) just after the stressor application, which is 4–5 times the standard deviation of the assay.

### Effect of Stress on Learning Performances

Irrespective of the experimental group, the number of successes (distal responses toward the rewarded cone) significantly increased from the first block of the acquisition session to the last block of the reacquisition session (W, Z_SB_ = −3.15, P_SB_ <0.01, Z_NS_ = −3.67, P_NS_ <0.001, Z_SA_ = −3.18, P_SA_ <0.01).

#### Acquisition performances

Independently of their groups, the horses progressively learned that they had to touch a cone during distal indications. Indeed, the number of distal responses increased from the 1^st^ to the 3^rd^ blocks of acquisition session, in both SB and NS+SA groups (W, SB group: 1^st^ block vs. 3^rd^ block: Z = −2.6, P<0.01; 1st block vs. 2^nd^ block: Z = −1.9, P<0.05; 2^nd^ block vs. 3^rd^ block: W, Z = −1.6, P = 0.10; NS+SA group: 1^st^ block vs. 3^rd^ block: Z = −3.6, P<0.001; 1st block vs. 2^nd^ block: Z = −2.1 P<0.05; 2^nd^ block vs. 3^rd^ block: Z = −3.6, P<0.01).

**Figure 5:**
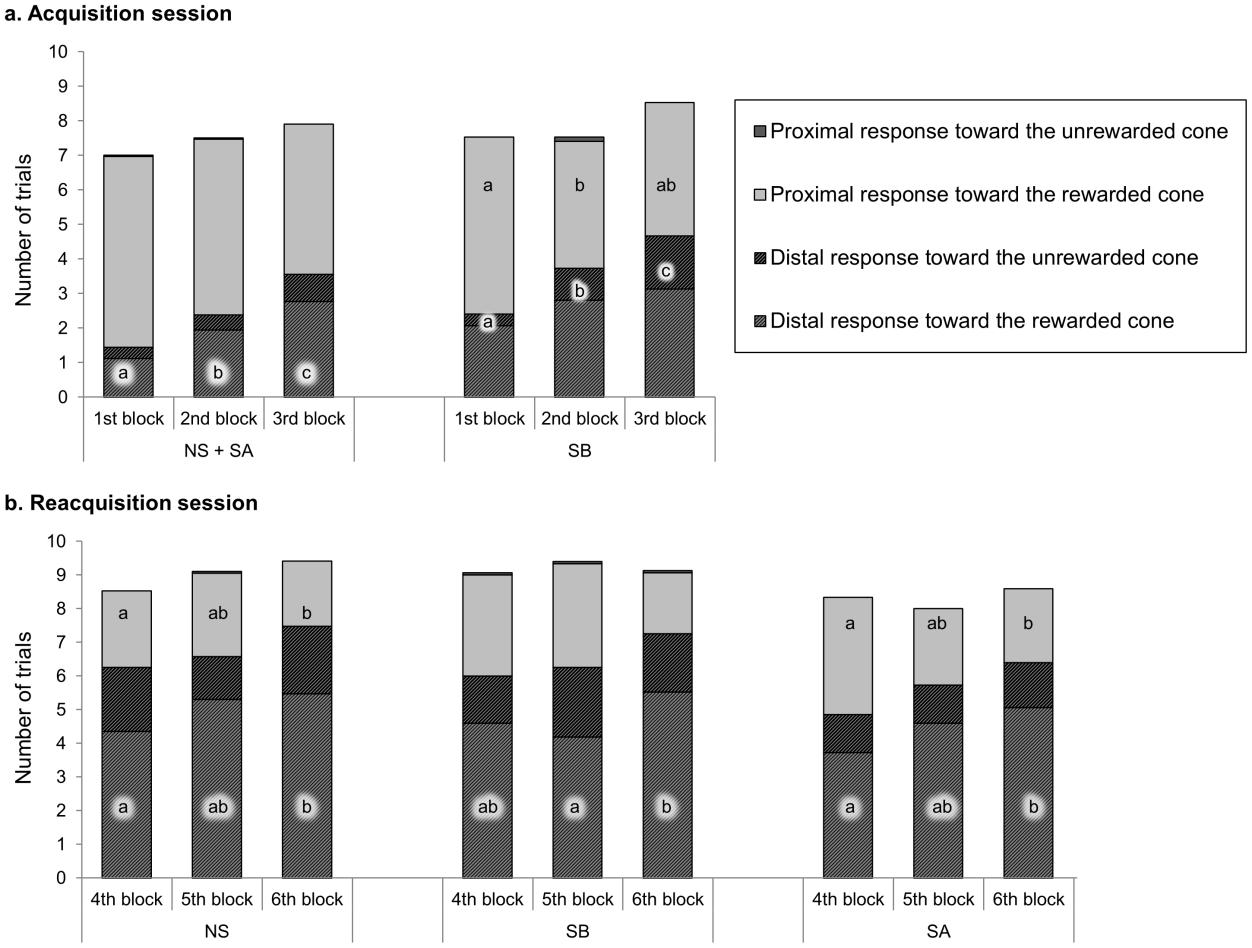
Distal and proximal responses displayed during acquisition and reacquisition sessions. Mean number of each type of distal and proximal responses displayed during the 1st, 2nd, and 3rd blocks of the acquisition session (a) and the 4th, 5th, and 6th blocks of the reacquisition session (b) by each group. Each block consisted of 10 trials. Intra-group differences between the blocks: a vs. b vs. c, P<0.05, Wilcoxon tests.

Among these distal responses, the number of successes tended to be higher in the SB group than in the NS+SA group during the 1^st^ block of acquisition (MW, U = 180.5, P = 0.09). This higher number of successes at the beginning of the session might explain why the number of successes was constant over the blocks in the SB group (F, ddl = 2, P>0.10), whereas it increased in the NS+SA group (W, 1^st^ block vs. 3^rd^ block: Z = −3.4 P<0.001; 1^st^ block vs. 2^nd^ block: Z = −2.2, P<0.05; 2^nd^ block vs. 3^rd^ block: Z = −2.8, P<0.01, **Fig. 5a**). Simultaneously, the number of proximal responses toward the rewarded cone of SB group significantly decreased from the 1^st^ to the 2^nd^ blocks of trials (W, Z = −2.1, P<0.05). Interestingly, the groups also differed in the number of distal responses toward the unrewarded cone. It was higher in the SB group than in the NS+SA group during the 2^nd^ block (MW, U = 94, P<0.05) and tended to be higher during the 3^rd^ block of the acquisition session (MW, U = 180, P = 0.09). In addition, it significantly increased from the 1^st^ block to the 3^rd^ block of trials in the SB group (W, 1^st^ block vs. 2^nd^ block: Z = −2.1, P<0.05; 1^st^ block vs. 3^rd^ block: Z = −2.3, P<0.05), but not in the NS+SA group (F, ddl = 2, P>0.10).

In summary, all the horses progressively learned to use distal cues to solve the task. Interestingly, SB horses tended to perform better than NS+SA horses at the beginning of the acquisition.

#### Retrieval performances

In order to assess retrieval performances, the percentage of horses that succeeded in the last trial of the acquisition session was compared with the percentage that succeeded in the first trial of the reacquisition session. These percentages did not vary significantly in any of the groups (%_Acquisition_ and %_Reacquisition,_ McNemar, SB: 33.4%, 40.0%, Q = 0.2, P>0.10; NS: 15.8%, 42.1%, Q = 2.8, P>0.10; SA 20.0%, 26.7%, Q = 0.2, P>0.10). The percentages of horses that successfully passed the first trial of the reacquisition session did not differ between the three groups of horses (2I, ddl = 2, P>0.10).

#### Reacquisition performances

**Figure 6:**
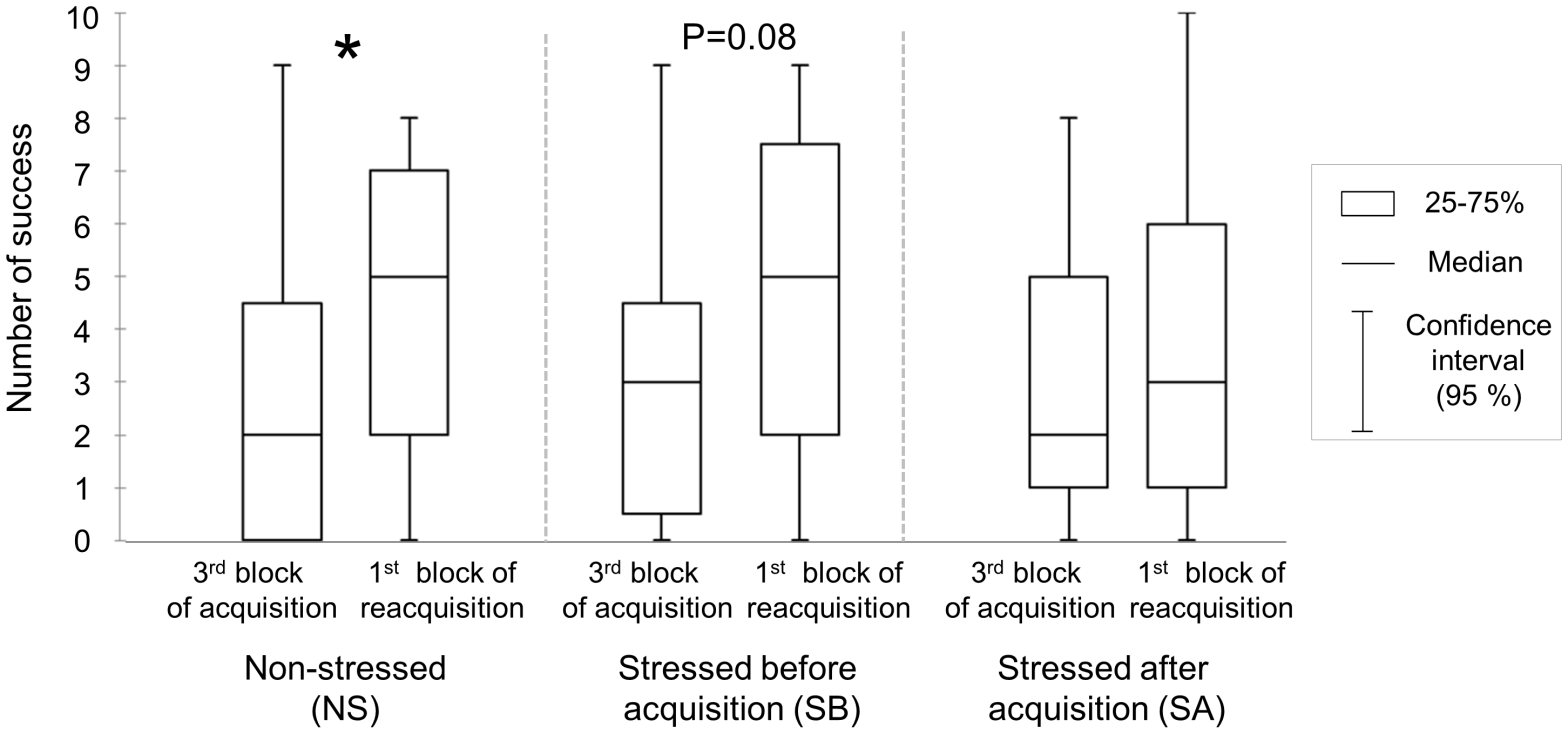
Distal successes at the end of acquisition session and at the beginning of reacquisition session. Boxplots of the numbers of distal successes displayed during the last 10 trials of the acquisition session and the first 10 trials of the reacquisition session. The number of distal successes tended to increase in SB horses and significantly increased in NS horses. No significant change was observed in SA horses. Wilcoxon test, *P<0.05.

In order to assess the reacquisition performances, we compared the number of distal successes in the last 10 trials of the acquisition session with the number of distal successes in the first 10 trials of the reacquisition session. In the NS group, performances were significantly improved from the end of the acquisition to the beginning of the reacquisition sessions (W, Z = −2.08, P<0.05, **Fig. 6**), suggesting a good reacquisition of the task. In the SB group, performances only tended to be improved (W, Z = −1.74, P = 0.08), whereas in the SA group, performances did not vary (W, Z = −1.39, P>0.10), suggesting lack of improvement between acquisition and reacquisition sessions.

During the reacquisition session (Fig. 5b), the number of distal responses increased or tended to increase in all the groups. It increased from the 4^th^ to the 6^th^ block in the NS group (W, 4^th^ block vs. 6^th^ block: Z = −2.7, P<0.01, 5^th^ block vs. 6^th^ block: Z = −1.7, P = 0.09), the SB group (W, 4^th^ block vs. 6^th^ block: Z = −1.7, P = 0.09, 5^th^ block vs. 6^th^ block: Z = −1.7, P = 0.09), and the SA group (W, 4^th^ block vs. 6^th^ block: Z = −2.4, P<0.05, 4^th^ block vs. 5^th^ block: Z = −1.7, P = 0.09). Among these distal responses, the number of successes also significantly increased over the blocks in all the groups: from the 4^th^ to the 6^th^ block in both the NS group (Z = −2.5, P = 0.01) and SA group (Z = −2.0, P<0.05), and from the 5^th^ to the 6^th^ block in the SB group (Z = −2.2, P<0.05). In parallel with this increase in successful trials, the number of proximal responses toward the rewarded cone decreased in both NS and SA groups from the 4^th^ block to the 6^th^ block of trials (W, NS horses: 4^th^ block vs. 6^th^ block: Z = −2.2, P<0.05; 5^th^ block vs. 6^th^ block: Z = −1.7, P = 0.09; SA horses: 4^th^ block vs. 6^th^ block: Z = −1.8, P = 0.07; 5^th^ block vs. 6^th^ block: W, Z = −2.0, P<0.05). No difference between the three groups was observed for any type of response (KW, ddl = 2, P>0.10).

### Behaviors Recorded during Acquisition and Reacquisition Sessions

#### Acquisition session

During the whole acquisition session, SB group explored the cones significantly more than the NS+SA group (M_SB_ = 3 (1.25–4.75), M_NS+SA_ = 1.5 (0–4), MW, U = 93.5, P<0.05). The percentage of horses that expressed snorting and startled reactions was significantly higher in the SB group than in the NS+SA group (Snorting: %_SB_ = 60%, %_NS+SA_ = 5.8%, 2I, ddl = 1, P<0.01; Startled reactions: %_SB_ = 26.7%, %_NS+SA_ = 2.9%, 2I, ddl = 1, P<0.05). However, the SB and the NS+SA groups did not differ in glancing at the experimenter (M_SB_ = 9 (4.75–13.5), M_NS+SA_ = 8 (3–13), MW, P>0.10), presenting alert posture (%_SB_ = 86.7%, %_NS+SA_ = 85.9%, 2I, ddl = 1, P>0.10), and blowing (%_SB_ = 33.3%, %_NS+SA_ = 17.6%, 2I, ddl = 1, P>0.10).

#### Reacquisition session

During the whole reacquisition session, the percentage of horses that exhibited an alert posture was significantly higher for the SB and SA groups than for the NS group (%_SB_ = 86.7%, %_SA_ = 80%, %_NS_ = 47.3%, 2I, ddl = 2, P<0.01). The SB, NS, and SA groups did not differ significantly in exploring the cones (M_SB_ = 4 (1.25–9), M_NS_ = 8 (2.25–15.75), M_SA_ = 5 (2–8.75), KW, ddl = 2, P>0.10), glancing at the experimenter (M_SB_ = 4 (2–7), M_NS_ = 4 (2–6.75), M_SA_ = 3.5 (2–6), KW, ddl = 2, P>0.10), snorting (%_SB_ = 20%, %_NS_ = 5.3%, %_SA_ = 13.3%, 2I, ddl = 2, P>0.10), or blowing (%_SB_ = 6%, %_NS_ = 20%, %_SA_ = 13.3%, 2I, ddl = 2, P>0.10).

### Influence of Temperament on Learning Performances

Significant correlations between variables of temperament and number of successes are summarized in **Table 1**. During the acquisition session, no significant correlation was found between the number of successes and the behavioral characteristics related to temperament in the NS+SA group (P>0.10). In the SB group, the number of successes in acquisition was positively and significantly correlated with the amount of trotting measured over all the temperament tests (P<0.05). During the reacquisition session, no significant correlation was found between the number of successes and the behavioral characteristics related to temperament of the NS group (P>0.10). In the SB group, the number of successes during the reacquisition session tended to be positively correlated with the amount of trotting measured over all the tests (P = 0.07). In the SA group, it was negatively and significantly correlated with the number of startled reactions over all the tests (P<0.01) and with the number of neighs during the social isolation test (P<0.01), and tended to be negatively correlated with the time taken to start eating again during the surprise test (P = 0.07). No other variables of temperament were significantly correlated with the successes during the entire acquisition or entire reacquisition sessions (P>0.10).

**Table 1:** Spearman correlations between temperament and the number of distal successes during acquisition and reacquisition sessions.

		Number of distal success				
		Acquisition session		Reacquisition session		
Temperamentdimension	Temperamentvariable	SBhorses	NS+SAhorses	SBhorses	NShorses	SAhorses
Fearfulness	Number of startledresponse	n.s.	n.s.	n.s.	n.s.	**r_s_ = **−**0.74, P<0.01**
	Eating latency duringsurprise test	n.s.	n.s.	n.s.	n.s.	*r_s_ = *−*0.50, P = 0.07*
Gregariousness	Number of neighsduring social isolation test	n.s.	n.s.	n.s.	n.s.	**r_s_ = **−**0.67, P<0.01**
Locomotor activity	Amount of trotting	**r_s_ = 0.61, P<0.05**	n.s.	*r_s_ = 0.48, P = 0.07*	n.s.	n.s.

Only variables showing a tendency (italics) or significant correlations (bold) are presented. In SB horses, the number of distal successes was significantly correlated during the acquisition session and tended to be correlated during the reacquisition session, with a temperament variable related to locomotor activity. In the SA horses, the number of distal successes during the reacquisition session was negatively correlated with temperament variables related to fearfulness and gregariousness. No significant correlation was noticed in the non-stressed horses. “n.s.” indicates an absence of significant correlation (P>0.10).

## Discussion

The present study shows that learning performances varied according to the exposure to stressors. Horses that were stressed before acquisition (SB group) tended to perform more successes at the beginning of the acquisition session than non-stressed horses did (NS+SA group). Eight days later, during the reacquisition session, contrary to NS animals, SA horses, and, to a lesser extent, SB horses, did not significantly improve their performance between acquisition and reacquisition sessions. Temperament influenced learning performances, but only when acquisition or reacquisition performances were affected by stress.

### Evolution of Learning Performances

In all experimental groups, the number of successes (distal responses toward the rewarded cone) increased from the first block of the acquisition session to the last block of the reacquisition session, showing that the individuals made progress. However, the number of successes remained relatively low over the blocks, showing that the task was difficult and that not all the individuals succeeded during the task. The difficulty of the task was not affected by a lack of motivation for food, nor by a difficulty to perform the act of touching the cone since the animals responded in at least 70% of the trials from the beginning of the acquisition with proximal and distal indications. The use of distal indications was not likely a reason for the difficulty of the task since previous studies suggest that horses are spontaneously able to use distal cues to localize food [34], [35]. Therefore, the difficulty of the task probably came from learning an association between distal cues, instrumental action, and reward. This finding is in accordance with McKinley and Sambrook [36], who also showed that forming an association between distal cues and an operant response (to search for food under a reversed bucket) could not be solved spontaneously by most of the horses. This difficulty induced a huge variability of performances across individuals, leading this task well adapted for studying the factors of variability of learning performances such as stress and temperament.

### Effect of Stress on Learning Performances

During the first block of acquisition session, stressed horses (SB group) tended to perform more successes with distal indications than non-stressed horses (NS+SA group), suggesting a positive effect of stress on performances. Several behavioral and physiological parameters have confirmed a state of stress in SB horses: they expressed more startled reactions and snorting, and showed higher salivary cortisol levels than the other horses. We suspect that this state of stress might have rendered the horses of the SB group more active at the beginning of the acquisition session, and thus made them more inclined to touch the cones and might explain why their performances were enhanced. This higher tendency for touching the cones might also explain the higher level of exploration of the cones between the inter-trial intervals observed in SB horses. However, the enhancement of learning performances was only temporary, since no difference was noticed during the second and third blocks of trials. Whether a longer or a deeper state of stress would be more efficient remains to be tested.

In contrast with the positive effect on acquisition performance, stress appears to impair the reacquisition processes. Indeed, SA horses did not show any significant improvement in performances from the end of the acquisition session to the beginning of the reacquisition session, unlike NS horses. The performances of SB horses only tended to increase and were then of an intermediate level. Since both SB and SA groups exhibited similar cortisol concentrations 30 min after the end of the acquisition session, these results suggest that an increased level of cortisol post-acquisition may be detrimental for further performances. This negative effect of stress cannot be attributed to an impairment of retrieval processes, since we did not find any significant variation in the number of horses that succeeded in the last trial of the acquisition session and the first trial of the reacquisition session in the SB, SA, and NS horses. Rather, we hypothesize that horses associated the stress applied before or after acquisition with the context of learning. Then, when replaced in the same context eight days later, this association might have induced a state of stress in SB and SA horses that impaired reacquisition of the task. Both SA and SB horses were more numerous than NS horses to exhibit alert postures during the reacquisition session, which are indicators of stress. This state of stress might have shifted the attention of the SA horses, and, to a lesser extent, the attention of the SB horses, from the learning task and impaired then the reacquisition performances [2], [37]–[39]. Interestingly, the effect of stress on reacquisition performances was stronger in SA horses than in SB horses and suggests that the same stressor, inducing the same increase in cortisol concentrations, is more deleterious for further performances when it occurs after the acquisition, rather than before. Thus, the association between the stressor and the learning context might be stronger when the stressor occurred after, because, in this last case, the learning-context might predict the subsequent occurrence of the stress episode. In accordance with this view, classical Pavlovian conditioning studies demonstrated that the association between an unconditioned stimulus and a conditioned stimulus is stronger if the unconditioned stimulus occurs after the conditioned stimulus (forward conditioning) rather than before (backward conditioning) [40]–[42].

To sum up, during the acquisition session, the state of stress of SB horses was induced directly by the exposure to the stressor just before the task and this state of stress tended to improve performances. On the contrary, during the reacquisition session, the state of stress might have been induced by a context-stress association in the SA and SB horses, and this state of stress impaired or tended to impair reacquisition performances.

### Effect of Stress on the Way to Respond

In addition to the effect of stress on performances, stress also had a significant impact on the behavior of the SB horses during the acquisition session. Indeed, stressed horses (SB) exhibited more distal responses toward the unrewarded cone than non-stressed horses (NS+SA) did. We hypothesize that the SB horses touched the unrewarded cone more because they made faster decisions during the trials, even before receiving the information necessary to correctly localize the rewarded cone. A state of stress is known to increase the speed of decision making [2], [43]. For instance, Keinan et al. [44] showed that stressed humans responded faster than non-stressed humans did during a cognitive task, even before they received all the information necessary to answer correctly. We did not find this difference of distal responses toward the unrewarded cone between the SB, SA, and NS horses during the reacquisition session. This may suggest that stress induced by a context-stress association does not affect decision-making processes, unlike stress induced by direct exposure to stressors.

### Temperament as an Amplifier of the Effect of Stress on Learning Performances

The influence of temperament dimensions on performances differed depending on the session, the presence of the stressor, and its timing. During the acquisition session, no correlation was found between temperament and acquisition performances in non-stressed horses. By contrast, SB horses that performed the best presented the highest level of locomotor activity (amount of trotting). Horses with an active temperament might have responded to the stressor by an increase of their locomotor activity, and this higher activity might have been maintained during the acquisition session. Consequently, they might have been more inclined to move and act during the task, and thus, to succeed. These results are in accordance with the study of Lansade and Simon showing that a high level of locomotor activity has a positive effect on acquisition performances, but only in a stressful task [45].

The same correlation was found during the reacquisition session in the SB group: the most active horses tended to perform more successes. We hypothesize that these horses tended to perform the best during the reacquisition session because of their higher performance during the previous session. On the other hand, in the SA group, the least fearful and the least gregarious horses performed more successes. These horses might have been less affected by stressors that involved social isolation and fearful events [46], [47]. Consequently, their reacquisition performances might have been less affected by the negative effect of stress. This negative correlation between fearfulness and performances was found in SA horses, but not in SB horses. We hypothesize that the deleterious effect of the stress before acquisition on reacquisition performances was too mild to cause differences of performances related to the fearfulness dimension since stress before acquisition tended to impair reacquisition performances, but not to the extent that stress after acquisition did.

Like in the acquisition session, in the non-stressed group, no correlation between temperament and performances was found during the reacquisition session. Taking into account that fearfulness impairs reacquisition performances in case of stress after acquisition, all these results suggest that stress is necessary to reveal the influence of temperament and impairs more cognitive abilities in fearful horses than in less fearful horses. The present study constitutes the first evidence that stress modulates the influence of temperament on cognitive abilities in horses.
